# miR-345-5p regulates adipogenesis via targeting VEGF-B

**DOI:** 10.18632/aging.103649

**Published:** 2020-09-14

**Authors:** Xiaofeng Liu, Yang He, Zhaolan Feng, Jianjian Sheng, Aiwu Dong, Meiying Zhang, Lingling Cao

**Affiliations:** 1 Department of Endocrinology, The First Hospital of Jiujiang City, Jiujiang 332000, China; 2 Department of Endocrinology, Zhuhai People’s Hospital (Zhuhai Hospital affiliated with Jinan University), Zhuhai 519000, China; 3 The Second Affiliated Hospital of Nanchang University, Nanchang 330000, China

**Keywords:** adipocyte, adipogenesis, miR-345, vascular endothelial growth factor

## Abstract

Adipocyte differentiation involves a series of highly synergistic processes, including clone amplification, proliferation arrest, and terminal differentiation. However, the mechanisms that control these different steps remain unclear. Emerging studies support that miRNAs play an important role in regulating adipogenesis. In this study, we found that the expression of miR-345-5p decreased during adipogenic differentiation, and overexpression of miR-345-5p reduced lipid accumulation in adipocytes and the expression of adipocyte related genes essential to lipogenic transcription, fatty acid synthesis and fatty acid transport. In addition, miR-345-5p directly targeted the 3’UTR of vascular endothelial growth factor B, and miR-345-5p mimic inhibited the expression of vascular endothelial growth factor B at both mRNA and protein levels. In conclusion, our results demonstrate that miR-345-5p inhibits adipocyte differentiation via targeting vascular endothelial growth factor B.

## INTRODUCTION

Adipocyte differentiation is an important biological process in the development of adipose tissue. Adipocyte differentiation involves clone amplification, proliferation arrest, and terminal differentiation, and is largely controlled by a complex transcription cascade involving C/EBPα, C/EBPβ, C/EBPδ, PPARγ, E2F1 and 2F4 [1, 2]. However, the mechanisms that control adipocyte differentiation remain not fully understand. The mouse 3T3-L1 cell line is a common preadipocyte cell line that has been widely used to explore the molecular mechanism of adipocyte differentiation [3]. 3T3-L1 cells will stop growing due to contact inhibition and then re-enter the cell cycle under the action of the combinations of hormones including insulin, cAMP analogues and glucocorticoids [4, 5]. After clone expansion, the cells regain the proliferation and differentiate into mature adipocytes eventually [6, 7].

MicroRNAs (miRNAs) are endogenous non-coding RNAs (20-24 nucleotides long) and play an important role in many processes such as cell proliferation, differentiation and development [8, 9]. The major function of miRNAs is to inhibit translation and/or promote mRNA decay by binding to the 3 '-untranslation region of target gene [10–12]. Several studies have evaluated the expression profile of miRNAs during adipocyte differentiation. For example, Esau et al. showed that miR-143 level was elevated and contributed to adipocyte differentiation [13–16]. Kajimoto et al. cloned 65 miRNAs from pre/post-differentiated 3T3-L1 cells and 21 miRNAs were found to be up- or down-regulated during differentiation [17]. Recently, Guo et al, reported that miR-345-5p was differentially expressed in undifferentiated human adipose-derived stem cells and differentiated adipocyte cells [18]. However, the role of miR-345-5p in regulating adipogenesis remains unexplored.

Therefore, in this study we aimed to elucidate the mechanism by which miR-345-5p regulates adipogenesis using 3T3-L1 preadipocytes as the model. We demonstrated that the level of miR-345-5p was down-regulated during adipocyte differentiation. Overexpression of miR-345-5p in 3T3-L1 preadipocytes impaired adipocyte differentiation, and inhibited the expression of adipocytic marker genes. Furthermore, miR-345-5p inhibited adipocyte differentiation by targeting the 3’UTR of vascular endothelial growth factor B (VEGF-B) to suppress its expression.

## RESULTS

### Downregulation of miR-345-5p during adipocyte differentiation

**Figure 1:**
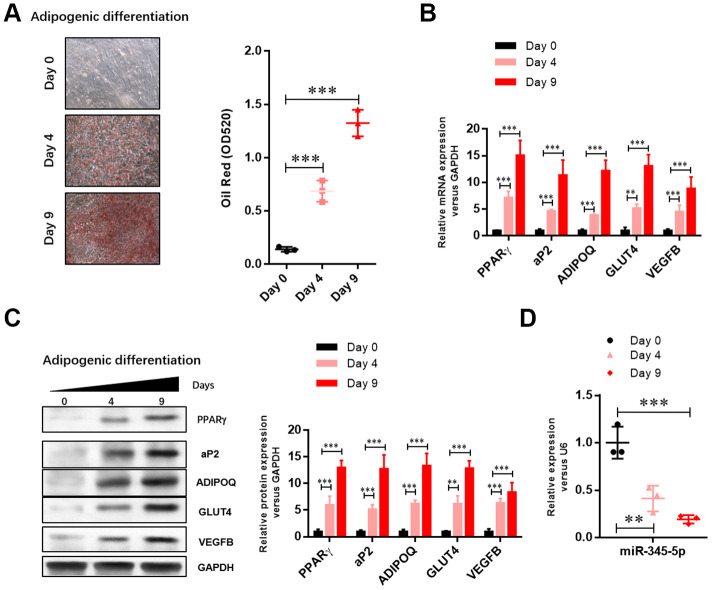
**The level of miR-345-5p was significantly downregulated during 3T3-L1 cell differentiation.** (**A**) Oil Red O staining of lipid droplets of 3T3-L1 cells at day 0, 4, and 9 during adipocyte differentiation. (**B**) Real-time PCR analysis of adipocyte-specific genes (PPARg, FABP4, aP2,ADIPOQ, GLUT4 and VEGF-B) in 3T3-L1 cells during adipocyte differentiation. (**C**) Western blot analysis of adipocyte-specific genes (PPARg, FABP4, aP2, ADIPOQ, GLUT4 and VEGF-B). (**D**) Real-time PCR analysis of miR-345-5p during adipocyte differentiation. *P<0.05, **P<0.01, ***P<0.00 versus Day 0 group.

To investigate the role of miR-345-5p in adipocyte differentiation, we evaluated the expression of miR-345-5p during 3T3-L1 differentiation. Lipid accumulation during 3T3-L1 cell differentiation into adipocytes was confirmed by Oil Red O staining (Figure 1A). The upregulation of adipocyte-specific genes was confirmed by real-time PCR (Figure 1B) and Western blotting (Figure 1C). Furthermore, quantitative real-time PCR showed that the level of mature miR-345-5p significantly decreased after the induction of adipocyte differentiation, and maintained at low level during adipogenesis (Figure 1D). These results suggest that miR-345-5p is downregulated during 3T3-L1 preadipocyte differentiation.

### miR-345-5p inhibited adipocyte differentiation

**Figure 2:**
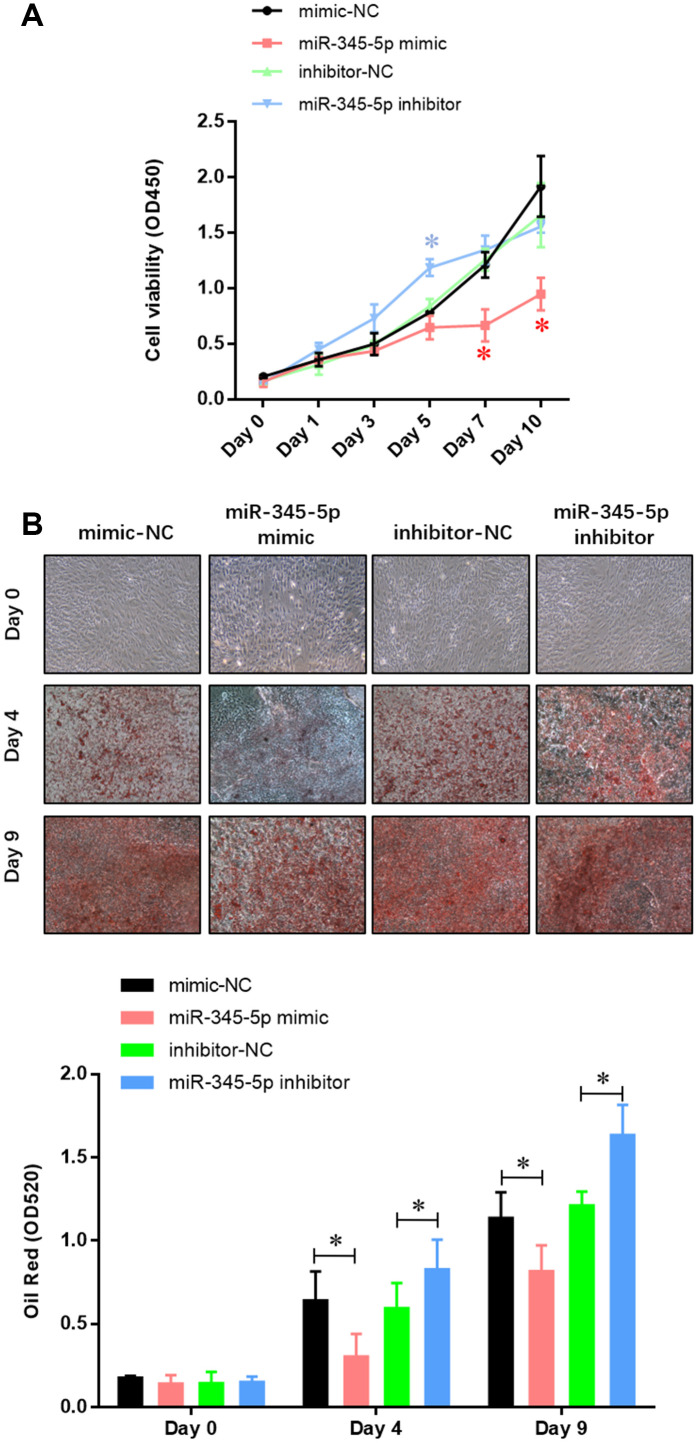
**miR-345-5p repressed 3T3-L1 preadipocytes differentiation.** (**A**) CCK-8 assay of the proliferation of 3T3-L1 cells. (**B**) Oil Red staining of lipid droplet formation of 3T3-L1 cells at day 0, 4, and 9 during adipocyte differentiation. *P<0.05 versus control group.

**Figure 3:**
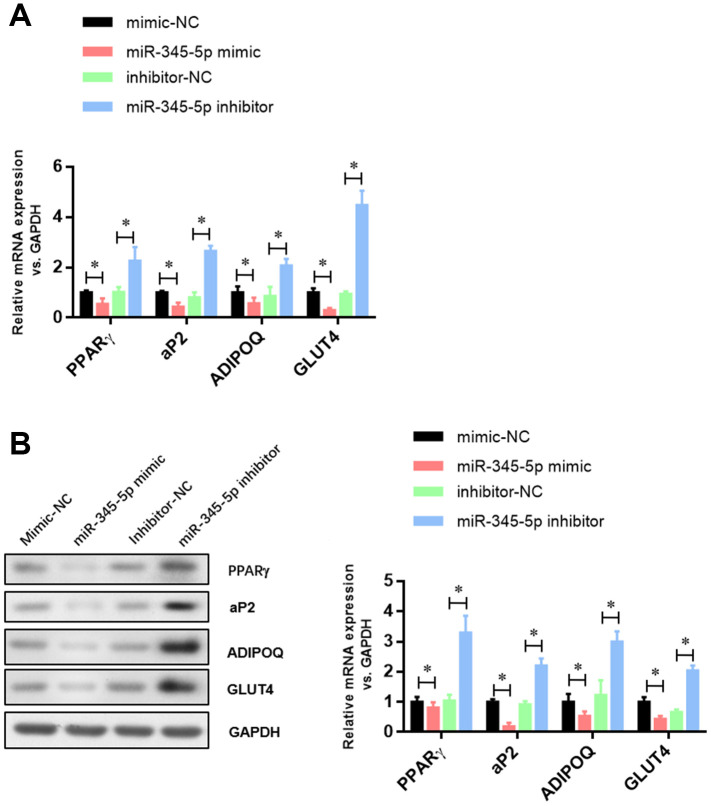
**The expression of adipocyte-specific genes was repressed by miR-345-5p.** (**A**) Real-time PCR analysis of adipocyte-specific genes (PPARg, FABP4, aP2, ADIPOQ, GLUT4 and VEGF-B) in 3T3-L1 cells during adipocyte differentiation. (**B**) Western blot analysis of adipocyte-specific genes (PPARg, FABP4, aP2, ADIPOQ, GLUT4 and VEGF-B). *P<0.05 versus control group.

To confirm the role of miR-345 in adipocyte adipogenesis, 3T3-L1 preadipocytes were transfected with miR-345-5p mimic/inhibitor or negative control (NC) before the induction of adipocyte differentiation. We found 100-fold increase in mature miR-345-5p level in 3T3-L1 cells transfected with miR-345-5p mimic (Figure 2A). miR-345-5p mimic strongly suppressed 3T3-L1 proliferation and differentiation into mature adipocytes (Figure 2B). Consistently, miR-345-5p inhibitor dramatically enhanced the number and proliferation of Oil Red O positive cells (Figure 2B). Furthermore, real-time PCR (Figure 3A) and Western blot analysis (Figure 3B) showed that compared with NC group, the expression of PPARγ, aP2 and adiponectin significantly decreased in cells transfected with miR-345-5p mimic, but increased in cells transfected with miR-345-5p inhibitor. Taken together, these results demonstrate that miR-345-5p could suppress 3T3-L1 preadipocyte differentiation.

### miR-345-5p targeted 3′ UTR of VEGF-B and suppressed its expression

**Figure 4:**
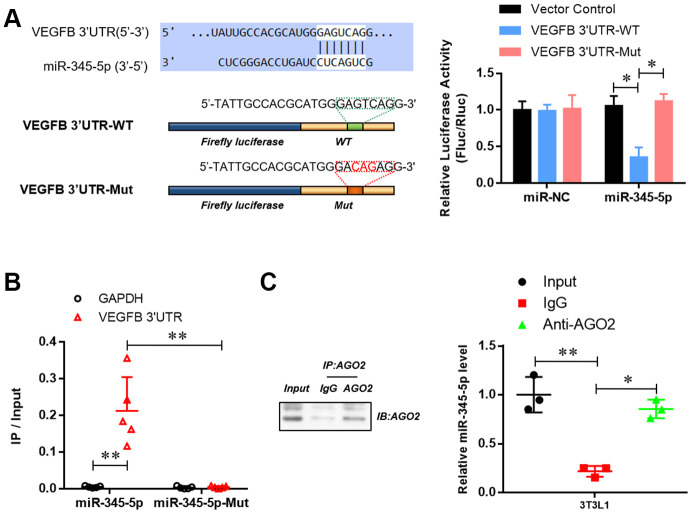
**miR-345-5p directly targeted 3′ UTR of VEGF-B.** (**A**) The dual-luciferase reporter assay of the binding of 3 'UTR of VEGFB and mir-345-5p. (**B**) RNA Pull-down assay of the specific binding of miR-345-5p to 3 'UTR of VEGFB. (**C**) AGO2 RNA-IP assay of the enrichment of mir-345-5p in AGO2 complex. *P<0.05 versus control group.

To reveal the underlying mechanisms by which miR-345-5p suppresses adipocyte differentiation, we went on to identify potential targets of miR-345. VEGF-B, a key component of insulin resistance signaling, was predicted as a potential target by Star Base, TargetScan, miRDB, and miRanda analysis. To verify that VEGF-B is a target of miR-345-5p, we constructed dual-luciferase report plasmids harboring the sequences of wild-type or binding site mutant (GTC to ACG) 3′ UTR of mouse VEGF-B (Figure 4A). As shown in Figure 4B, co-transfection of miR-345-5p mimic significantly decreased luciferase activity in cells transfected with wild-type VEGF-B 3′ UTR reporter (psiCHE-VEGF-B 3′ UTR-WT), compared with NC group. In contrast, no decrease in luciferase activity was observed in cells co-transfected with empty vector or mutant reporter (psiCHE-VEGF-B 3′ UTR-mut), suggesting that VEGF-B is a direct target of miR-345-5p.

RNA pull-down assay further revealed the enrichment of VEGF-B 3’UTR in miR-345-5p-captured fraction, compared with that of miR-345-5p mutation, in which the binding site of miR-345-5p for VEGF-B 3’UTR was disrupted (Figure 4C). Furthermore, AGO2 immunoprecipitation showed that endogenous VEGF-B 3’UTR could pull-down miR-345-5p (Figure 4D).

**Figure 5:**
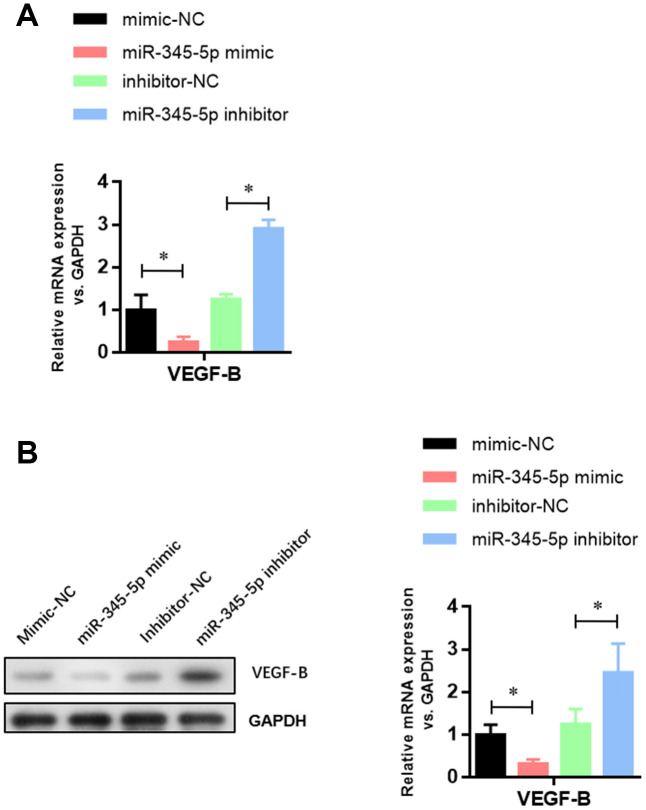
**miR-345-5p suppressed the mRNA and protein levels of VEGF-B.** (**A**) Real-time PCR analysis of VEGF-B mRNA in 3T3-L1 cells. (**B**) Western blot analysis of VEGF-B protein in 3T3-L1 cells. *P<0.05 versus control group.

To investigate whether miR-345-5p can regulate the expression of VEGF-B, VEGF-B mRNA and protein levels were measured in 3T3-L1 cells transiently transfected with miR-345-5p mimic by qPCR and Western blotting, respectively. We found that miR-345-5p inhibited endogenous VEGF-B mRNA (Figure 5A) and protein (Figure 5B) expression in 3T3-L1 preadipocytes. Collectively, these results indicate that VEGF-B is a direct target of miR-345-5p.

## DISCUSSION

The differentiation of preadipocytes into mature fat cells requires a series of highly precise regulation in gene expression [4]. Although a transcription factor cascade has been identified that contributes to adipocyte differentiation, the underlying mechanisms that control the different phases of adipogenesis are not completely understood. Herein, we demonstrated that miR-345-5p inhibits 3T3-L1 preadipocyte differentiation through targeting the 3’ UTR of VEGF-B and suppressing the expression of VEGF-B.

VEGF-B is highly expressed in cells with metabolic activity, such as brown adipocytes, skeletal myocytes, myocardiocyte and pancreatic β-cells [19, 20]. The interaction of VEGF-B with VEGF receptor 1 and its co-receptor neuropilin induces the expression of vascular-specific fatty acid transport protein 3 (FATP3) and FATP4 [21]. VEGF-B knockout mice are healthy and fertile, but exhibit decreased fatty-acid uptake and lipid deposition in muscles [19]. In contrast, cardiac specific up-regulation of VEGF-B causes ceramide accumulation in the heart, which eventually leads to dysfunction of mitochondrial quality control [21].

Although VEGF-B has great potential for improving tissue vascularization, its function is limited to cardiac tissue, which has the highest endogenous expression of VEGF-B. Hagberg et al., proposed that VEGF-B induces fatty acid transport across the endothelium in brown adipose tissue, skeletal muscle, heart, and that blockade of VEGF-B may be a novel treatment for type 2 diabetes [22]. Inhibition of VEGF-B was shown to prevent lipid deposition, increase peripheral glucose uptake, maintain fasting and postprandial glucose levels, and improve glucose tolerance and insulin sensitivity [23–26]. In this study, we demonstrated that miR-345-5p inhibited endogenous VEGF-B expression via targeting its 3’UTR. Taken together, these observations suggest that targeting miR-345-5p/VEGF-B axis could be a novel approach to prevent glucose resistance and pathological lipid deposition.

In summary, our study reveals a novel mechanism that miR-345-5p suppresses adipocyte differentiation, at least in part by inhibiting the expression of VEGF-B and adipogenic genes. Therefore, miR-345 and its targets may potentially regulate pathological progression of obesity-related diseases.

## MATERIALS AND METHODS

### Cell culture

3T3-L1 cells were cultured in high glucose with l-glutamine DMEM (Thermo Fisher, Carlsbad, CA, USA) with 10% fetal bovine serum (FBS), 100 U/ml penicillin, and 100 μg/ml streptomycin (all from Thermo Fisher), and maintained in a 5% CO_2_ humidified atmosphere. For adipocyte differentiation, cells were incubated in conditional medium (5 μg/ml insulin, 0.5 mM 3-isobutyl-1-methylxanthine, and 1 μM dexamethasone) 2 d after the cells reached confluence. The culture medium was replaced with DMEM containing 10% FBS and 5 μg/ml insulin 48 hours later. The culture medium was replaced every 2 days until the preadipocytes differentiated into mature adipocytes around 9 days later.

### Cells viability assay

Differentiated 3T3-L1 cells were seeded in poly-D-lysine coated 96-well culture plates at and cultured for 24 h. At the end of treatment, cells were incubated with WST-8 (5 mg/ml, Abcam, Cambridge, MA,, USA) at 37°C for 3 h. Absorbance at 450 nm wave length was measured using a VICTOR3 spectrophotometer (Perkin Elmer Italia, Milano, Italy).

### Oil red O staining

3T3-L1 cells were fixed with 10% formaldehyde in PBS at 37°C for 90 min, washed with sterile water and stained with 200 μL of oil red O solution at 37°C for 2 h. After the staining, cells were incubated with 200 μL of isopropanol, and the absorbance at 520 nm wave length was measured using a VICTOR3 spectrophotometer (Perkin Elmer Italia, Milano, Italy).

### Quantitative real-time PCR

RNAs were extracted from cells and quantified by RT-qPCR using SYBR green assay on an ABI 7900HT system. All primers were designed and synthesized by Sangon Biotech (Shanghai, China): PPARg CAAGAATACCAAAGTGCGATCAA, GAGCTGGGTCTTTTCAGAATAATAAG; FABP4 CCTGGTGATGTCCGACCTG, TCCTCCATTAGGAACTCTCACAC; ADIPOQ TATTCGGACAAATACGACGACG, GGTTCCTCCATTCAGATTCAGAC; GLUT4 CAGCTCTCAGGCATCAAT, TCTACTAAGAGCACCGAG; VEGFB ACCAGAAGAAAGTGGTGCCATG, TGAGGATCTGCATTCGGACTTG; GAPDH TGCTGAGTATGTCGTGGAGTCT, ATGCATTGCTGACAATCTTGAG. miR-345-5p levels were measured by real-time PCR using the TaqMan MicroRNA Assay Kit (Applied Biosystems, Foster City, CA, USA).

Quantification of the expression of target genes in the samples was presented as the difference of reaction cycle thresholds (Ct) between GAPHD and each of the target genes (2^-ΔCt^).

### Western blot analysis

Cells were lysed in RIPA solution (Beyotime, China), and proteins isolated from cells were quantitated by bovine serum albumin method. Total 20 μg protein samples were separated by 12% sodium dodecyl sulfate-polyacrylamide gel and transferred to polyvinylidene difluoride membrane (Millipore, USA). The membrane was blocked at 4°C for 2 h with 5% goat serum, incubated with antibodies (1:1,000) against PPARg (Abcam, USA), FABP4 (Abcam, USA), ADIPOQ (Abcam, USA), GLUT4 (Abcam, USA), VEGF-B (Abcam, USA) and GAPDH (Simo Biotech, Shanghai, China) for 2 h at 25°C, followed by incubation with secondary antibody (Cell Signaling, Danvers, MA, USA) for 1 h at 25°C. The ECL Chemiluminescence reagents (Millipore, USA) were used to visualize the protein bands and the quantity-one software was used to quantify them.

### Luciferase reporter assay

The wild-type and miR-345-5p binding site mutant (GTC to CAG) of 3′UTR of VEGF-B were sub-cloned into psiCHECK-2 vector (Promega, USA). 3T3-L1 cells were seeded in 24-well plates and transfected with miR-345-5p mimic or NC along with psiCHECK-2 vector by using Lipofectamine 3000 Transfection Reagent. Dual-Glo Luciferase Assay System (Promega, USA) was used to measure luciferase activity 48 h after transfection.

### Statistical analysis

Quantitative results were represented as mean ± SEM and analyzed by GraphPad Prism 6 software. Statistical differences between two groups were assessed by Student’s multiple t-tests. Significant difference was considered at *P* < 0.05.
